# Recurrent Painful Ophthalmoplegic Neuropathy in an Adult Patient: A Case Report With Literature Review

**DOI:** 10.7759/cureus.25297

**Published:** 2022-05-24

**Authors:** Shehbaz M Ansari, Sumeet G Dua, Mustafa Mafraji

**Affiliations:** 1 Department of Diagnostic Radiology and Nuclear Medicine, Rush University Medical Center, Chicago, USA

**Keywords:** cranial nerve enhancement, third nerve palsy, diplopia, recurrent painful ophthalmoplegic neuropathy, ophthalmoplegic migraine

## Abstract

Recurrent painful ophthalmoplegic neuropathy (RPON), previously known as ophthalmoplegic migraine, is a rare disease that predominantly affects children. Recurrent episodes of ocular cranial nerve paresis with ipsilateral headache characterize this disorder. Diagnosis is mainly clinical with imaging being used as an adjunct. The pathophysiology of the disease is unknown. We present here a case of RPON in a 50-year-old female presenting with multiple episodes of headache and diplopia with associated transient thickening and enhancement of the ipsilateral oculomotor nerve on magnetic resonance imaging (MRI).

## Introduction

Recurrent painful ophthalmoplegic neuropathy (RPON), previously known as “ophthalmoplegic migraine,” is a rare disorder characterized by recurrent episodes of one or more ocular cranial nerve paresis with ipsilateral headache [1,2]. The estimated annual incidence is 0.7 per million [3]. The terminology was updated, and the diagnostic criteria were laid down by the third edition of the International Classification of Headache Disorders, 2018 (ICHD-3) [1]. The oculomotor is the most affected nerve, with mydriasis and ptosis being the common symptoms [3]. Although it commonly affects children with an average age of eight years, the disorder has also been reported in older adults [2,4].

## Case presentation

A 50-year-old right-handed female with a past medical history significant for hypertension, well-controlled type II diabetes mellitus, and chronic obstructive pulmonary disease presented with intermittent episodes of diplopia. She was taking 500 mg of metformin and 5 mg of amlodipine for diabetes and hypertension, respectively. Her hemoglobin A1c group (HbA1c) has been normal for the last two years at least.

The first episode of diplopia was two years prior when she had daily episodes of diplopia and periorbital pain, worse on waking up and in the evening. She was diagnosed with oculomotor nerve palsy. She was treated with steroids resulting in gradual improvement in the symptoms. The diplopia eventually resolved three months later. She was symptom-free for six months. She then presented to our hospital with daily episodes of headache, posterior in nature. This was followed by recurrence of diplopia and pain with eye movement, a couple of weeks later. Diplopia was most prominent on looking up and to the right. The symptomatology was similar to her prior episode nine months earlier, except that the pain was worse. She denied any fever, weakness, numbness, unintentional weight loss, or history of autoimmune diseases. On examination, she had a non-reactive left pupil.

**Figure 1:**
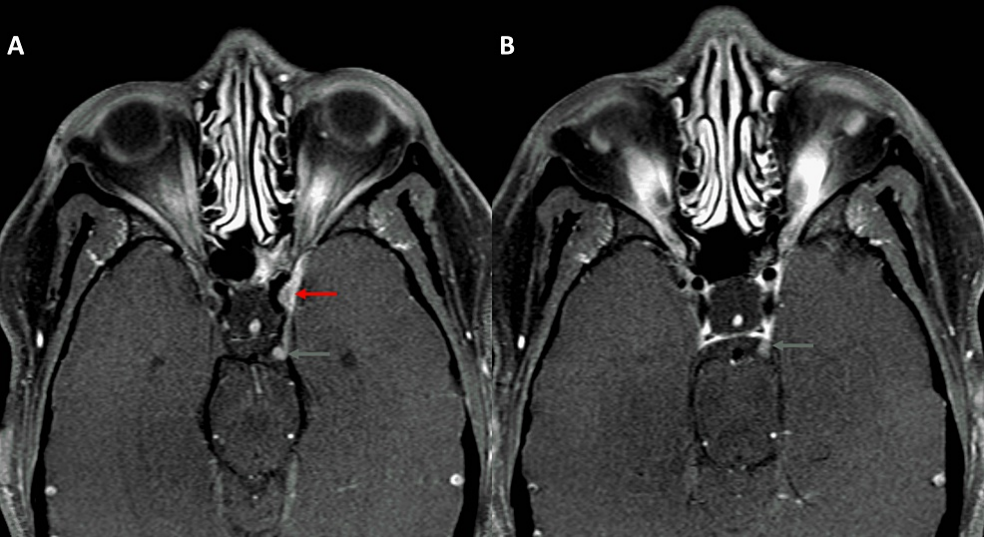
Postcontrast axial T1-weighted images (T1WI) showing nodular thickening in the region of the cavernous (red arrow in A) and cisternal segment (green arrows in A and B) of the left oculomotor nerve Note that nodular thickening of the cisternal segment was visible on two consecutive images (both A and B).

**Figure 2:**
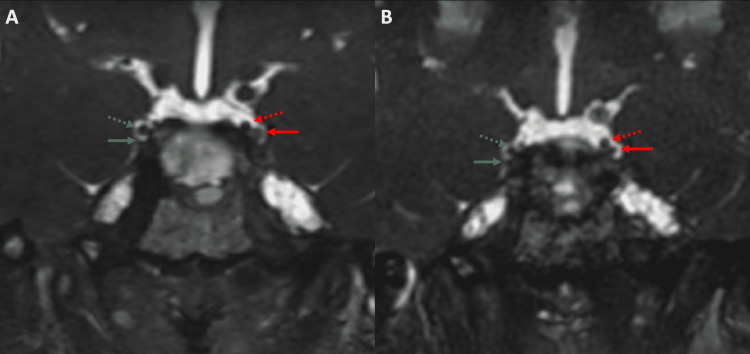
3D CISS sequence obtained along with the images in Figure 1 (A) and 10 months later (B) The left oculomotor nerve in A (solid red arrow) is thicker than the adjacent posterior communicating artery, PcomA (dotted red arrow). The right oculomotor nerve and PcomA are marked for comparison (solid and dotted green arrow, respectively). After 10 months (B), with the resolution of the thickening of the left oculomotor nerve, it becomes smaller than the adjacent PcomA. CISS: Constructive interference in steady-state; PcomA: Posterior communicating artery.

Magnetic resonance imaging (MRI) of the brain and orbit revealed enhancing nodules in the lateral wall of the cavernous sinus and basal cistern (Figure 1). These enhancing nodules were confirmed to be representing nodular thickening of the left oculomotor nerve on three-dimensional (3D) constructive interference in steady-state (CISS) sequence (Figure 2). The right third cranial nerve was unremarkable as were bilateral intraorbital soft tissues. No other parasellar or posterior fossa abnormality was noted on imaging.

Differential diagnoses of nerve sheath tumor, lymphoid/granulomatous disease, and atypical infections were considered. Infection was thought to be less likely in the absence of any systemic signs. She had a workup for sarcoidosis including computed tomography (CT) of the chest and a gallium scan. CT chest revealed nonspecific cystic changes, presumed to be due to heavy marijuana use. Gallium scan showed nonspecific uptake in bilateral parotid and submandibular glands. Parotid gland biopsy was negative for malignancy or granulomatous infection. Extensive cerebrospinal fluid (CSF) analysis including culture and cytology was negative. Serum angiotensin-converting enzyme (SACE) and antineutrophil cytoplasmic antibodies (ANCA) levels were normal.

**Figure 3:**
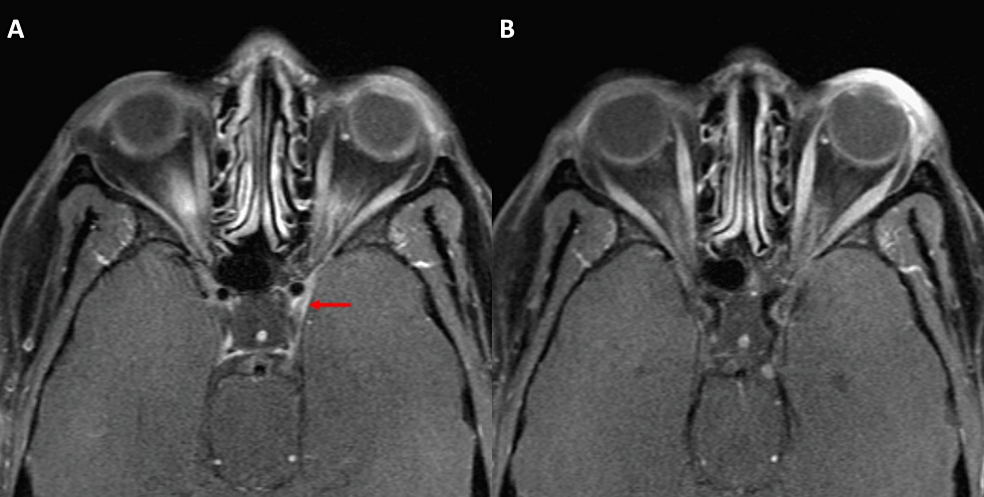
MRI orbit obtained four months later revealed a marked reduction in the thickening of the cavernous segment of the left oculomotor nerve (red arrow in A) and mild reduction in the cisternal segment (green arrow in B).

**Figure 4:**
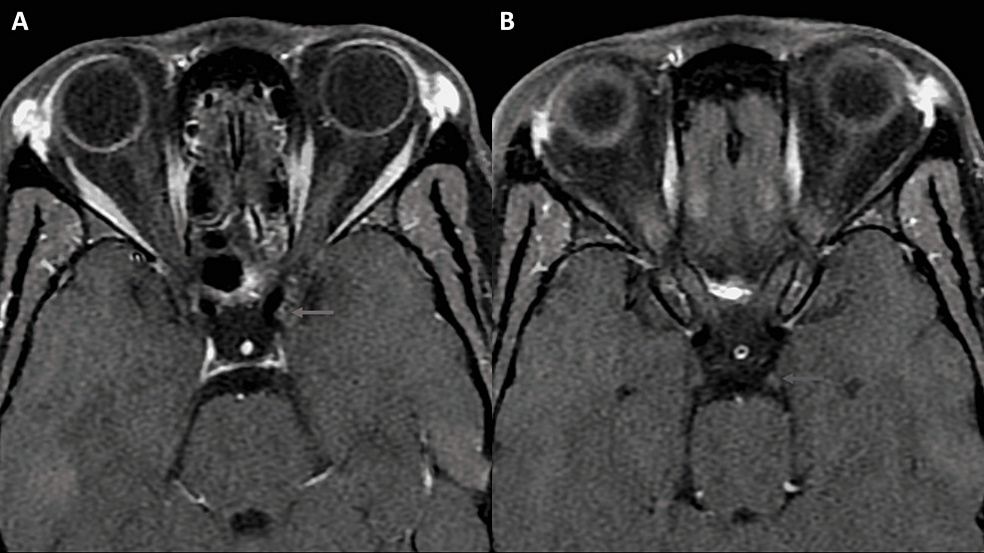
MRI orbit obtained 10 months after the presentation shows near-complete resolution of the thickening and enhancement of both cavernous and cisternal segments (arrows in A and B).

She was given 20 mg of oral prednisone twice daily. Her diplopia showed gradual improvement with near-complete resolution after four months. Imaging obtained at this time revealed a mild reduction in the size of the nodular enhancement (Figure 3). Follow-up imaging six months later showed that the nodular thickening had almost resolved (Figure 4). Due to multiple self-resolving episodes of diplopia with headache and typical imaging findings, a diagnosis of RPON was made. The patient is currently on follow-up.

## Discussion

Third nerve palsy can be due to an abnormality affecting different locations along the oculomotor nerve pathway ranging from involvement of one or more oculomotor nuclei by abnormalities such as infarct and demyelinating disease to orbital apex involvement in Tolosa-Hunt syndrome [5]. Isolated involvement of cisternal and/or cavernous segment of the nerve, as in our case, can be seen with varied etiologies like metabolic diseases, neoplasm, inflammatory disease, or infectious processes (Table 1) [5,6].

**Table 1:** Main differential diagnosis of the oculomotor nerve pathologies CSF: Cerebrospinal fluid.

Disease category	Relevant diagnostic tools	Expected findings
Metabolic	Imaging studies and clinical history	Negative imaging findings and positive relevant past medical history
Neoplasm	Imaging studies	Enhancing lesion with progressive interval growth on follow-up imaging
Systemic inflammatory disease	Imaging studies and/or laboratory investigations	Stigmata of other organ system involvement on imaging and/or positive autoantibodies
Infection	CSF analysis	Positive CSF culture and/or serology

Metabolic diseases such as diabetes are the most common causes of oculomotor palsy [6]. Isolated diabetic third nerve palsy is either unremarkable on imaging or shows brainstem lesions [7]. Multiple self-resolving episodes typically do not occur. Other common etiologies of isolated third nerve palsy such as aneurysm and extra-axial hematoma were ruled out on imaging.

Enhancing third nerve on imaging can be seen with neoplastic, inflammatory, or infectious causes [8]. A neoplasm can directly affect the nerve such as in schwannoma or secondarily due to perineural spread from head and neck cancers [9,10]. But neoplasm causing isolated third nerve involvement rarely resolves with steroids. Inflammatory diseases such as neurosarcoidosis and granulomatosis with polyangiitis are known to affect the oculomotor nerve [5]. However, there are no associated findings on the CT chest, and the absence of symptoms and signs of other organ system involvement go against these etiologies. Viral, bacterial, and fungal infections have been reported with third nerve palsy [11-13]. Recurrent self-resolving episodes, absence of infectious focus anywhere in the body, and negative CSF culture suggest otherwise. Hence, as per the criteria laid down by ICHD-3 (Table 2) [1], a diagnosis of RPON was established.

**Table 2:** RPON diagnostic criteria by the third edition of the ICHD-3 RPON: Recurrent painful ophthalmoplegic neuropathy; ICHD-3: International Classification of Headache Disorders, 2018.

Diagnostic criteria
A. At least two attacks fulfilling criterion B
B. Both of the following: Unilateral headache AND ipsilateral paresis of one, two, or all three ocular motor nerves
C. Orbital, parasellar, or posterior fossa lesion has been excluded by appropriate investigation
D. Not better accounted for by another ICHD-3 diagnosis

The RPON is a diagnosis of exclusion; no other etiology should better account for the findings [1]. Also, RPON is mainly a clinical diagnosis with imaging being an adjunct [3]. The RPON is a rare disorder occurring predominantly in children [3]. It is associated with recurrent headaches and ophthalmoplegia [3]. The pathophysiology of RPON is poorly understood [8]. The various hypotheses proposed include chronic inflammatory demyelination, autoimmune process, reversible ischemic state of the nerve, simple nerve compression, and even oculomotor schwannoma with palsy due to the release of chemicals from the tumor causing inflammation, demyelination, and remyelination [8,14]. Pathological evaluation is usually limited due to the self-resolving, benign nature of the episodes. Hence, verification of these hypotheses is difficult [15].

Imaging finding reported with RPON includes thickening and enhancement of one or more of the ocular cranial nerves, most commonly the oculomotor nerve [3,4,8,16-18]. The nerve thickening can be smooth or nodular [3,4,8,16,17]. Follow-up imaging is important. The nerve enhancement and thickening are known to improve as the clinical symptoms resolve [3]. Enhancement and thickening of the fourth and the sixth nerves can also be seen, but less commonly [16,18].

The nerve thickening can be better evaluated and monitored on the 3D CISS sequence because of its superior spatial resolution, as shown in our case. The advantage of using these sequences for assessing cranial nerve pathologies is well known [19]. However, to the best of our knowledge, this is the first case of RPON, which was evaluated using a 3D CISS sequence. This sequence is called a different name by different vendors, viz., fast imaging employing steady-state acquisition (FIESTA) by GE, fast imaging with steady-state free precession (FISP) by Siemens, balanced fast field echo (FFE) by Philips, and steady-state free precession (SSFP) by Toshiba. Because of uncertain pathophysiology, the management of RPON is not well-defined. The use of prednisolone, acetaminophen, non-steroidal anti-inflammatory drugs (NSAIDs), ergotamine, triptans, and even cyproheptadine hydrochloride have been tried with variable success [15].

## Conclusions

RPON is a rare disorder diagnosed based on specific history aided by the presence of nerve thickening and enhancement on imaging. Imaging also helps rule out other pathologies. Although common in children, it can also be seen in adults. 3D CISS sequence MRI imaging may be a better way to monitor nerve thickening as has been the case with other common cranial nerve pathologies.
